# Association between sleep duration and quality and depressive symptoms among university students: A cross-sectional study

**DOI:** 10.1371/journal.pone.0238811

**Published:** 2020-09-11

**Authors:** Wang Li, Jianjun Yin, Xianfeng Cai, Xin Cheng, Yongxiang Wang

**Affiliations:** 1 Department of Physical Education, Huaiyin Institute of Technology, Huaian, People’s Republic of China; 2 Department of Physical Education, Guangdong University of Finance & Economics, Guangzhou, People’s Republic of China; University of Toronto, CANADA

## Abstract

Sleep duration and quality have several effects on human health. Some previous studies have shown an association between sleep duration and quality and mental health, but the results of those studies were inconsistent. Additionally, studies on sleep duration and its impact on depressive symptoms specifically among young Chinese adults are limited. Hence, this study aimed to investigate the association between duration and quality of sleep and depressive symptoms among Chinese university students. We designed a cross-sectional study comprising 9,515 Chinese university students. Sleep duration and quality were assessed using a self-reported questionnaire. Depressive symptoms were assessed based on the Self-rating Depression Scale score. Logistic regression models were used to analyze the association between sleep duration and quality and depressive symptoms. The results showed that good sleep quality was associated with a lower prevalence of depressive symptoms. In the final adjusted model, the odds ratios and 95% confidence intervals for the prevalence of depressive symptoms in those with poor sleep quality compared with those with normal and good sleep quality were 0.88 (0.77, 1.01) and 0.82 (0.81, 0.96), respectively (p for trend = 0.014). Moreover, short sleep duration was associated with a higher prevalence of depressive symptoms based on the crude model and final adjusted model (p for trend = 0.048 and 0.042, respectively). Poor sleep quality and short sleep duration were associated with a higher prevalence of depressive symptoms in this study population. These results suggest that reduced sleep duration and quality may be risk factors for mental health disorders among university students.

## Introduction

Depressive symptoms are common mental disorders associated with significant personal, social, and economic effects [1, 2]. Depressive symptoms also lead to negative health outcomes such as functional impairment [3], cardiovascular disease [4], and even suicidal deaths [5]. A higher prevalence of depressive symptoms is found not only in middle-aged and elderly individuals, but also among university students [6]. According to previous studies, depressive symptoms could affect the academic performances and physical functions of university students [7]. Depressive symptoms are commonly found among university students because they live independently and experience changes in their lifestyles and relationships with others. Considering that young adulthood is an important period linking adolescence and adulthood, the health status developed during this period may significantly affect an individual’s life. Thus, maintaining optimal mental health is crucial during this period.

Sleep is an active and periodic biological state that involves a homeostatic process; it is important to physical and mental health [8]. Thus, a good sleep state is important for maintaining good health. Epidemiological studies have indicated that sleep duration is associated with the risks of hypertension, diabetes, obesity, and even mortality [9–12]. Poor sleep quality has been reported to be associated with a higher risk of memory impairment and poorer cognitive function [13]. Additionally, some previous studies have shown that short sleep duration and poor sleep quality are associated with a higher prevalence of depressive symptoms in adolescents and adults. However, evidence on this association specifically among university students (young adults) is limited, especially the association between sleep duration and depressive symptoms. Considering that the lifestyle and sleep habits of university students are different from those of other populations, it is important to evaluate whether sleep duration and quality are associated with depressive symptoms among university students. Thus, in this study, we aimed to investigate the association between sleep duration and quality and depressive symptoms among a sample of Chinese university students. We hypothesized that students who slept for a shorter duration or who had poorer sleep quality would be more likely to have depressive symptoms than those who slept for longer durations or who had better sleep quality.

## Materials and methods

### Participants

This was a cross-sectional study. A total of 9,980 university students aged 16–27 years were enrolled from Huaiyin Normal University in the Jiangsu Province of China between June 2018 and October 2018. The study participants were recruited from a physical health examination for university students. A survey was conducted using an anonymous, self-administered questionnaire and performed in the measurement room before the examination. Participants were excluded if their data for sleep (n = 285), depressive symptoms (n = 112), family income (n = 65), and physical activity (n = 3) were missing. Finally, data from 9,515 participants were used in the analysis. Participants in this study were only from one university in a city of China; thus, the findings of this study may present just an area in China, and not represent all Chinese university students. This study was approved by the Ethics Committee of the Huaiyin Institute of Technology. All participants provided written informed consent.

### Sleep assessment

Sleep duration and quality were evaluated using a self-reported questionnaire comprising questions about sleep conditions that have been used in a previous study [14]. We assessed sleep duration by asking the participants the following questions: “For how many hours did you usually sleep at night in the past month?” The responses for sleep duration were as follows: “<5 h,” “5–6 h,” “6–7 h,” “7–8 h,” “8–9 h,” and “>9 h.” We subsequently divided these responses into the following three categories: <7 h, 7–8 h, and >8 h. Additionally, students were asked to rate their difficulties with initiating and maintaining sleep using the following five-point scale: 1, < 1 day per month; 2, 1–3 days per month; 3, 4–7 days per month; 4, 8–15 days per month; and 5, ≥ 16 days per month. A score of 1 or 2 indicated good sleep quality, 3 indicated normal sleep quality, and 4 or 5 indicated poor sleep quality.

### Assessment of depressive symptoms

We used the Chinese version of the Self-rating Depression Scale (SDS) to assess whether participants had depressive symptoms. The SDS is widely used for measuring the severity of depression; it is a self-reporting instrument comprising 20 questions with good internal consistency and validity that encompasses most Diagnostic and Statistical Manual IV criteria for major depression [15, 16], and has been used in many studies on Chinese populations [17, 18]. The SDS index score ranges from 20 to 80. In this study, a cutoff score of 45 was used to define depressive symptoms [17, 19].

### Covariates

Body mass index (BMI) was calculated as weight/height^2^ and subsequently categorized into the following three groups according to the guidelines for the Chinese population: <18.5, underweight; 18.5–24.0, normal weight; and >24.0, overweight [20]. Physical activity was assessed using the International Physical Activity Questionnaire. Total physical activity was calculated as follows: metabolic equivalents (METs) × hour/week [21]; physical activity was then divided into the following three categories: low, middle, and high. Family income was divided into three categories: ≤5000 yuan/month (low income), 5001–8000 yuan/month (middle income), and >8000 yuan/month (high income). Information on the sex, grade, tobacco smoking, and alcohol drinking status of the participants were obtained by conducting a questionnaire survey.

### Statistical analyses

Statistical analyses were performed using the International Business Machines Corporation (IBM) Statistical Package for the Social Sciences version 24.0 (Statistical Product and Service Solutions) for Windows (IBM Inc., New York, USA). All tests were two-tailed, and the significance level (α) was set at p <0.05. Sleep duration and quality were considered as independent variables, and depressive symptoms were considered as dependent variables. Differences between sleep categories for the proportional variables were examined using logistic regression analysis. Multivariate logistic regression analysis was used to determine the association between sleep duration and quality and depressive symptoms in unadjusted and adjusted models. To adjust for potentially confounding variables, Model 1 was adjusted for sex, grade, and BMI; Model 2 was adjusted for variables in Model 1, along with physical activity, family income, and smoking and drinking status. Additionally, considering that sleep duration may influence sleep quality, we adjusted sleep duration to assess the association between sleep quality and depressive symptoms in Model 2.

## Results

Of the 9,515 (men: 5,670 [59.6%]) eligible participants, 1,847 (19.4%) had depressive symptoms (SDS score >45). Table 1 shows the characteristics of the participants according to depressive symptoms. No statistically significant association was observed between the depressive symptoms and sociodemographic factors.

**Table 1:** Characteristics of participants with and without the depressive symptoms.

	Total	Nondepressed	Depressed	*p* value^1^
	n (%)	n (%)	n (%)	
Gender				0.428
Male	5670 (59.6)	4554 (59.4)	1116 (60.4)	
Female	3845 (40.4)	3114 (40.6)	731 (39.6)	
BMI (kg/m^2^)				0.475
< 18.5	1480 (15.6)	1191 (15.5)	289 (15.6)	
18.5–24	5462 (57.4)	4423 (57.7)	1039 (56.3)	
> 24	2573 (27.0)	2054 (26.8)	519 (28.1)	
Grade				0.947
First year	2965 (31.2)	2398 (31.3)	567 (30.7)	
Second year	2619 (27.5)	2113 (27.6)	506 (27.4)	
Third year	2329 (24.5)	1870 (24.4)	459 (24.9)	
Fourth year	1602 (16.8)	1287 (16.8)	315 (17.1)	
Physical activity				0.146
High	3163 (33.2)	2517 (32.8)	646 (35.0)	
Middle	3166 (33.3)	2581 (33.7)	585 (31.7)	
Low	3186 (33.5)	2570 (33.5)	616 (33.4)	
Smoking habits				0.531
Non-smoker	8323 (87.5)	6699 (87.4)	1624 (87.9)	
Smoker	1192 (12.5)	969 (12.6)	223 (12.1)	
Drinking habits				0.482
everyday	497 (5.2)	410 (5.3)	87 (4.7)	
occasionally	2499 (26.3)	2003 (26.1)	496 (26.9)	
Non-drinker	6519 (68.5)	5255 (68.5)	1264 (68.4)	
Family income				0.296
Low	3495 (36.7)	2827 (36.9)	668 (36.2)	
Middle	2370 (24.9)	1884 (24.6)	486 (26.3)	
High	3650 (38.4)	2957 (38.6)	693 (37.5)	

^1^ Obtained using χ2 test for proportional variables.

Participants’ characteristics according to sleep duration and quality are presented in Table 2. Compared with the category of short sleep duration (<7 hours), long sleep duration was significantly associated with a lower percentage of drinking every day (p for trend = 0.003). The proportion of men, participants with BMI >24, and participants who drank occasionally were significantly higher in the good sleep quality categories (p for trend = 0.009, 0.009, and 0.031, respectively). Additionally, the proportion of participants who drank daily was significantly lower in the good sleep quality categories (p for trend = 0.030).

**Table 2:** Characteristics of the participants according to categories of sleep duration and quality.

	Sleep duration (hours/day)			p for trend^1^
	<7	7–8	>8	
n	2615	5112	1788	
Sex (male; %)	59.5	58.7	62.1	0.139
BMI (kg/m^2^)				
< 18.5	15.9	16.0	13.9	0.108
18.5–24	58.4	56.7	58.1	0.684
> 24	25.7	27.4	28.0	0.077
Grade (%)				
First year	30.9	31.6	30.4	0.836
Second year	28.0	27.1	28.2	0.990
Third year	24.2	24.3	25.4	0.408
Fourth year	16.9	17.1	15.9	0.496
Physical activity (%)				
High	33.5	32.9	33.7	0.951
Middle	32.8	34	31.9	0.656
Low	33.7	33.1	34.4	0.701
Smoker (%)	12.4	12.4	13.0	0.594
Drinking status (%)				
Drinking everyday	5.7	5.6	3.5	0.003
Drink occasionally	26.1	25.5	28.6	0.111
Non-drinker	68.1	68.9	67.8	0.937
Family income (%)				
Low	36.4	36.5	37.8	0.365
Middle	25.4	25.0	23.8	0.261
High	38.2	38.4	38.4	0.919
	Sleep quality			
	Poor	Normal	Good	
n	1801	5112	2602	
Sex (male; %)	57.3	59.5	61.3	0.009
BMI (kg/m^2^)				
< 18.5	15.5	16.1	14.5	0.265
18.5–24	59.4	57.0	56.8	0.126
> 24	25.2	26.9	28.7	0.009
Grade (%)				
First year	32.9	30.5	31.2	0.300
Second year	26.9	27.9	27.2	0.932
Third year	23.4	24.5	25.3	0.158
Fourth year	16.8	17.1	16.4	0.659
Physical activity (%)				
High	32.8	33.4	33.2	0.853
Middle	32.9	33.9	32.2	0.529
Low	34.3	32.6	34.6	0.657
Smoking status (%)				
Smoker	12.2	12.0	13.0	0.392
Drinking status (%)				
Drinking everyday	5.2	5.8	4.0	0.030
Drink occasionally	27.2	24.4	29.3	0.031
Non-drinker	67.6	69.7	66.7	0.314
Family income (%)				
Low	35.9	37.1	36.5	0.758
Middle	25.2	24.4	25.7	0.587
High	38.9	38.5	37.7	0.430

^1^ Obtained by using logistic regression analysis for variables of proportion.

Table 3 shows the odds ratios (ORs) and confidence intervals (CIs) for depressive symptoms according to sleep duration and quality categories. Compared to <7 hours sleep duration, 7–8 hours and >8 hours sleep duration categories were significantly associated with a lower prevalence of depressive symptoms, showing a clear inverse linear trend in the unadjusted model (p for trend = 0.048). In Model 1, this significant inverse association did not change, and the ORs for the occurrence of depressive symptoms in the three categories of sleep duration were as follows: 1.00 (reference), 0.97 (95% CIs, 0.86–1.09), and 0.85 (95% CIs, 0.72–0.99) (p for trend = 0.045). This significant inverse association remained unchanged even after adjusting for Model 2 (p for trend = 0.042). Regarding the adjusted association between sleep quality and depressive symptoms, in the unadjusted model, compared to the poor sleep quality category, the ORs and 95% CIs of the normal and good sleep quality categories were significantly associated with a lower prevalence of depressive symptoms (p for trend = 0.018). This significant inverse association did not change after adjusting for potential confounding factors in Models 2 and 3 (p for trend = 0.016 and = 0.014, respectively).

**Table 3:** Adjusted associations between sleep duration and quality and depressive symptoms.

	All subjects (n)	Depressive symptoms (n)	Unadjusted model	Model 1^3^	Model 2^4^
Sleep duration (hours/day)					
< 7	2615	527	1.00	1.00	1.00
7–8	5112	1005	0.97 (0.86, 1.09)^2^	0.97 (0.86, 1.09)	0.97 (0.86, 1.09)
> 8	1788	315	0.85 (0.73, 0.99)	0.85 (0.72, 0.99)	0.84 (0.72, 0.98)
p for trend^1^			0.048	0.045	0.042
Sleep quality					
Poor	1801	384	1.00	1.00	1.00
Normal	5112	985	0.88 (0.77, 1.01)	0.88 (0.77, 1.00)	0.88 (0.77, 1.01)
Good	2602	478	0.83 (0.72, 0.97)	0.83 (0.71, 0.96)	0.82 (0.71, 0.96)
p for trend^1^			0.018	0.016	0.014

^1^ Obtained by multiple logistic regression analysis.

^2^ Values represent adjusted odds ratios and parenthetical values represent 95% confidence intervals. (all such values).

^3^ Adjusted for sex, grade, and body mass index.

^4^ Adjusted for Model 1 plus drinking and smoking habits, physical activity, family income. Additionally adjusted using sleep duration for the relationship between sleep quality and depressive symptoms.

## Discussion

This study investigated the association between sleep duration and quality and depressive symptoms among university students. Although adjustments were made for a number of confounding factors, this study found that short sleep duration (<7 hours) was associated with a higher prevalence of depressive symptoms, while good sleep quality was inversely associated with depressive symptoms. This suggests that sleep duration and quality may be associated with the mental health of university students.

A previous study showed that, among Turkish university students, the rate of depressive symptoms was 27.1% [6]. Another study showed that the prevalence of depressive symptoms was 29.7% among Chinese university students [22]. However, we found that the rate of depressive symptoms in our study was 19.4%, which is lower than that reported in those previous studies, possibly because the method of assessment of depressive symptoms and participants were different. Moreover, our results are inconsistent with the results of an American study (rate of depressive symptoms was 85% among university students) and a Korean study (rate of depressive symptoms was 34.4% among young adults) [23, 24]. In contrast, the rate of depressive symptoms was lower than 10% in studies conducted among Chinese adolescents [25, 26]. Our findings suggest that higher prevalence of depressive symptoms among university students is still an increasing public health concern in China.

Consistent with the findings of our study, several studies on Chinese university students showed that poor sleep quality is associated with depressive symptoms, such as the study by Tao et al., which indicated that poor sleep quality positively correlated with depressive symptoms in 4,747 college students (OR: 4.97, 95% CI: 3.99–6.19) [27], and the study by Lau et al., which showed that poor sleep quality positively predicted higher levels of depressive mood in 1,628 Chinese university students [28]. Although, different assessment methods of depressive symptoms were used in these previous studies, our findings strengthened the evidence on the association between sleep quality and depressive symptoms in Chinese university students. In addition, a previous prospective longitudinal study also indicated that poor sleep quality can exacerbate the risk of postnatal depression in 228 perinatal women [29]. However, we could not find any previous study on the association specifically between sleep duration and depressive symptoms among young Chinese adults (age: 19–24 years). An American cross-sectional study comprising 1,258 rural adults suggested that short sleep duration (<7 hours per night) is positively associated with elevated depressive symptoms [30]. On the contrary, two cross-sectional studies comprising 150,053 Chinese adolescents (7th–12th grades) and 1,788 American adults (aged 19–89 years) indicated a U-shaped association between sleep duration and depressive symptoms [26, 31]; although the results of these two studies were inconsistent with the results of this study; nevertheless, all other studies showed that short sleep duration (<7 hours) is associated with a higher prevalence of depressive symptoms. Furthermore, a study investigated the association between sleep duration and quality and depressive symptoms among 1,992 Japanese university freshmen; although the methods and samples were different from those used in this study, the study is similar to our study. This study showed that poor sleep quality is significantly associated with a higher risk of depressive symptoms and suicidal ideation. Moreover, according to this study, individuals who slept for 7–8 hours at night had a lower prevalence of depressive symptoms than those who slept for less than 7 hours or more than 9 hours [32].

Although the mechanisms involved in the association between sleep duration and quality and depressive symptoms are unknown, there are some possible explanations. First, poor sleep quality has been shown to contribute to chronic inflammation [33], and it is associated with melatonin dysregulation [34], both of which are associated with the development of depressive symptoms and mood disorders [35, 36]. Second, individuals who sleep for a short duration may have insufficient rest and greater perceived stress severity [37], and perceived stress is reported as a risk factor for depressive symptoms [38]. Third, an animal study indicated that when subjected to chronic sleep restriction, neurotransmitter receptor systems were gradually altered in a manner similar to that in individuals diagnosed with major depression [39]. A study on human samples reported that university freshmen who carry two alleles of low-expressing polymorphism of the serotonin transporter gene reported a more depressed mood in the presence of a persistent pattern of short nocturnal sleep [40]. Fourth, both good sleep conditions and good mental health could be considered indicators of a healthy lifestyle. Thus, we hypothesized that a positive association might exist between sleep condition and mental health.

This study has several limitations that must be acknowledged. First, the data on sleep duration and quality were entirely assessed by self-evaluations, which may not reflect the real sleep quality/duration status and may cause errors. Second, because it was a cross-sectional study, it is difficult to draw conclusions about causality. Third, we did not use standardized scales to assess sleep duration and quality (e.g., Pittsburgh Sleep Quality Index) because of the limited survey time. Fourth, although a number of confounding factors were adjusted for analysis, we could not exclude the possibility that depressive symptoms are affected by other factors that correlate with sleep status. Fifth, a previous study indicated that approximately 1.5% of university students take hypnotic drugs [14]; however, we were not able to ask questions about medication/drug use. Finally, our data were obtained from one university; thus, they probably did not represent all Chinese university students.

This study found that short sleep duration and poor sleep quality were associated with a higher prevalence of depressive symptoms among Chinese university students. Our findings suggest that sleep status may have potential effects on the mental health of young adults and is also expected to provide important information to aid health education and preventive medicine. A randomized trial study is required to clarify causality.

## Supporting information

S1 File Questionnaire in Chinese. (DOCX)

S2 File Questionnaire in English. (DOCX)
