# Supplementary material for: Association between sleep duration and quality and depressive symptoms among university students: A cross-sectional study
Source: PLoS One. 2020 Sep 11;15(9):e0238811. doi: 10.1371/journal.pone.0238811 (PMC7485879; doi:10.1371/journal.pone.0238811)
Supplement: S1 File — (DOCX) [file pone.0238811.s001.docx]

**2018年度**

调 查 问 卷

◆请填写你的个人信息。

姓名 （ ）

学号 （ ）

手机 （ ）

住址

请您亲自认真填写此问卷。对于问卷内容不要做过深考虑，只需要对问题的字面意思做出直观回答就可以。

- **(回答选择题时请在选项上画◯) 注意一定不要漏填**

1. 性别： 1. 男 2. 女
2. 出生年月日： 年 月 日
3. 民族： 1. 汉族 2. 少数民族
4. 年级： 1. 一年级 2. 二年级 3. 三年级 4. 四年级
5. 居住方式： 1. 寝室 2. 家里 3. 租房 4. 其他
6. 你的家庭收入是多少？（父母收入总和） 元／月
7. 你吸烟吗？

1. 不吸 2. 曾经吸，但现在不吸 3. 吸烟

1. 你平时能够吸到二手烟吗？

1. 经常能 2. 偶尔能 3. 不能

1. 你饮酒吗？

1. 不喝 2. 每月1-2次 3. 每月3-4次 4. 每月4次以上

1. 在过去的30天以内你有因为喝醉酒而感到不适或呕吐吗？

1. 没有 2. 1-2次 3. 3次以上

1. **a**你在过去一年间有连续三个月以上，或者现在持续一个月以上的疼痛吗？

1. 有 2. 没有

**b**如果回答［有］，疼痛的部位是哪里？（请在疼痛部位画◯）

・头 ・颈部 ・胸 ・腹部 ・后背 ・腰 ・肩 ・肘 ・手

・股关节 ・膝盖 ・脚 ・其他（ ）

1. 你在过去的一个月当中平均每天刷几次牙？

1. 四次以上 2. 三次 3. 二次 4. 一次 5. 不到一次

1. 你在过去的一年里有过不小心摔倒的经历吗？

1. 有 （ 次） 2. 没有

1. 你与身边同学的关系如何

1. 融洽 2. 一般 3. 不融洽

1. 除住校时间外你与谁一起生活？

1. 父母亲 2. 父亲 3. 母亲 4. 其他

1. 你的家庭经济状况如何？

1. 非常好 2. 好 3. 一般 4. 不好

1. 与其他人相比你的吃饭速度如何？

1. 快 2. 普通 3. 慢

1. 过去的一个月里，每周有几次在晚上睡觉前2个小时内吃晚饭？

1. 每天 2. 六次 3. 五次 4. 四次 5. 三次

6 二次 7. 一次 8. 一次以下 9. 没有

1. 过去的一个月里，你每周吃早饭的次数。

1. 每天 2. 六次 3. 五次 4. 四次 5. 三次

6 二次 7. 一次 8. 一次以下 9. 不吃

1. 在过去的一个月里，你每周晚饭后吃零食的次数。

1. 每天 2. 六次 3. 五次 4. 四次 5. 三次

6 二次 7. 一次 8. 一次以下 9. 不吃

1. 在过去的一个月里，你每周自己一个人吃晚饭的次数。

1. 每天 2. 六次 3. 五次 4. 四次 5. 三次

6 二次 7. 一次 8. 一次以下 9. 没有

1. 除体育课外，你有参加校内或校外的体育社团吗？

1. 有 2. 没有

1. **a**你有一天三十分钟以上，每周三天以上的定期的运动习惯吗（不包含散步）？

1. 有（坚持了多久？ 年 月） 2. 没有

**b**回答［没有］的同学，你不运动的理由是什么（多项选择）？

1. 没有时间 2. 身体弱 3. 对体力没自信 4. 没有合适场所

5. 没朋友一起 6. 没人指导 7. 没钱 8. 没兴趣 9. 没理由

1. 你的家庭对体育运动的支出有规划吗？

1. 有 2. 没有

1. 你的家人（不包含自己）有运动习惯吗

1. 有 2. 没有

1. 你上学期的专业课成绩如何（还没有专业课的同学请回答综合成绩）？

1. 优 2. 良 3. 及格 4. 不及格

1. 身体活动量调查

（认真阅读下面问题后，在做回答。**※激烈的身体活动**是指身体感到非常疲惫，而且呼吸非常急促的活动，**适度的身体活动**是指身体感到稍有负荷，而且呼吸明显加快的活动。）

**1a**、 最近一周内，您有几天做了**剧烈的身体活动**（比如提重物、挖掘、有氧运动或是快速骑自行车等）？

◆　每周 天

◆　没有 →跳到问题2a

**1b、** 在这其中一天您通常会花多少时间在剧烈的体育活动上？

小时 分钟

**2a、** 最近7天内，您有几天做了**适度的身体活动**，（比如提轻的物品、以平常的速度骑车或打双人网球等）？请不要包括走路。

◆　每周 天

◆　没有 →跳到问题3a

**2b、** 在这其中一天您通常会花多少时间在适度的身体活动上？

小时 分钟

**3a、** 最近7天内，您有几天是步行，且一次步行至少10分钟？

◆　每周 天

◆　没有 →跳到问题4

**3b、** 在这其中一天您通常花多少时间在步行上？

小时 分钟

**4、** 最近七天内，每个工作日您有多久时间是坐着的？

小时 分钟

1. 下面一些问题是关于你最近一个月的睡眠状况，请按照实际情况回答。

a.近一个月，每夜通常实际睡眠几个小时？

(1) <5 小时 (2) 5-6 小时 (3) 6-7 小时

(4) 7-8 小时 (5) 8-9 小时 (6) >9 小时

b.近一个月，你有多少次感到入睡困难和不能保持良好睡眠

(1) < 1 天 (2) 1-3 天 (3) 4-7 天

(4) 8-15 天 (5) ≥ 16 天

1. 认真阅读下面问题，在最适合你现在的状态的栏里画◯，请回答全部问题。

|  | | 很少有 | 偶尔 | 经常 | 持续 |
| --- | --- | --- | --- | --- | --- |
| 1 | 我觉得闷闷不乐，情绪低沉 |  |  |  |  |
| 2 | 我觉得一天中早晨最好 |  |  |  |  |
| 3 | 一阵阵哭出来或觉得想哭 |  |  |  |  |
| 4 | 我晚上睡眠不好 |  |  |  |  |
| 5 | 我吃得跟平常一样多 |  |  |  |  |
| 6 | 我与异性亲密接触时和以往一样感到愉快 |  |  |  |  |
| 7 | 我发觉我的体重在下降 |  |  |  |  |
| 8 | 我有便秘的苦恼 |  |  |  |  |
| 9 | 心跳比平常快 |  |  |  |  |
| 10 | 我无缘无故地感到疲乏 |  |  |  |  |
| 11 | 我的头脑和平常一样清楚 |  |  |  |  |
| 12 | 我觉得经常做的事情并没有困难 |  |  |  |  |
| 13 | 我觉得不安而平静不下来 |  |  |  |  |
| 14 | 我对未来抱有希望 |  |  |  |  |
| 15 | 我比平常容易生气激动 |  |  |  |  |
| 16 | 我觉得做出决定是容易的 |  |  |  |  |
| 17 | 我觉得自己是个有用的人，有人需要我 |  |  |  |  |
| 18 | 我的生活过得很有意思 |  |  |  |  |
| 19 | 我认为如果我死了，别人会生活得更好 |  |  |  |  |
| 20 | 平常感兴趣的事我仍然感兴趣 |  |  |  |  |

1. 你对在学校的学习感到有压力吗？

1. 没有 2. 有一些压力 3. 压力非常大

1. 你有能使自己努力奋斗的目标吗？

1. 有 2. 没有

1. 你觉得自己活的有意义吗？

1. 有 2. 没有 3. 不知道

1. 在过去的一年当中你是否有过要自杀的想法？

1. 没有 2. 有过一次 3. 有过两次以上

1. 在过去的一年当中你是否尝试过自杀？

1. 没有 2. 尝试过一次 3. 尝试过两次以上

1. 你认为自己快乐吗？

1. 快乐 2. 不快乐

1. 你在过去一年当中有过几次感冒的经历？

1. 没有 2. 一次 3. 两次以上

- 体力测定结果

|  | 男生 | 女生 |
| --- | --- | --- |
| 身高（厘米） |  |  |
| 体重（公斤） |  |  |
| 肺活量（毫升） |  |  |
| 50米（秒） |  |  |
| 立定跳远（米） |  |  |
| 坐位体前屈（厘米） |  |  |
| 1000 米（男） |  |  |
| 800 米（女） |  |  |
| 引体向上（男） |  |  |
| 仰卧起坐（女） |  |  |
| 握力（公斤） |  |  |

谢谢你的合作！
