# Supplementary material for: Association between sleep duration and quality and depressive symptoms among university students: A cross-sectional study
Source: PLoS One. 2020 Sep 11;15(9):e0238811. doi: 10.1371/journal.pone.0238811 (PMC7485879; doi:10.1371/journal.pone.0238811)
Supplement: S2 File — (DOCX) [file pone.0238811.s002.docx]

**2018**

Questionnaire

◆Please fill in your personal information.

Name （ ）

ID number （ ）

Phone number （ ）

Address

Please fill in the questionnaire. You may just give your answers according to the literal meaning of the question.

- **(Check ◯ on the answer you have chosen) Just be careful not to miss out any question.**

1. Gender： 1. Male 2. Female
2. Data of Birth：Day Month Year
3. Nationality： 1. Han nationality 2. Minority
4. Grade： 1. Freshman 2. Sophomore 3. Junior 4. Senior
5. Mode of living： 1. Dormitory 2. Home 3. Rental house 4. Others
6. How about your family income? RMB/month
7. Do you smoke？

1. Never 2. Smoke previously 3. Yes

1. Do you have passive smoking at ordinary times?

1. Often 2. Occasionally 3. No

1. How often do you have a drink containing alcohol？

1. Never 2. 1-2 times/month 3. 3-4 times/month 4. More than 4 times/month

1. Have you felt uncomfortable or sick for drinking during the past thirty days?

1. No 2. Once or twice 3. Over three times

1. **a.** Have you suffered pains for over three consecutive months in the past year or for over one month at present?

1. Yes 2. No

**b.** If your answer is [Yes], where is the location of pain? (Check ◯ on the location of pain)

・Head ・Neck ・Chest ・Abdomen ・Back ・Waist

・Shoulder ・Elbow ・Hand ・Femoral Joint ・Knee ・Foot ・Others（ ）

1. How many times a day did you brush your teeth in the past month?

1. More than 4 2. Three 3. Two 4. One 5. Less than one

1. Have you been tumbled in the past year?

1. Yes （ times） 2. No

1. How is your relationship with your classmates?

1. Good 2. Not bad 3. Bad

1. Who do you live with?

1. Parents 2. Father 3. Mother 4. Other

1. How about your family economic conditions?

1. Very good 2. Good 3. Ordinary 4. Terrible

1. Compared to others, how quickly do you eat?

1. Fast 2. Normal 3. Slow

1. How many days a week did you have dinner 2 hours before sleep in the past month?

1. Everyday 2. Six days 3. Five days 4. Four days 5. Three days

6. Two days 7. One day 8. Less than one day 9. Never

1. How many days a week did you eat breakfast in the past month?

1. Everyday 2. Six days 3. Five days 4. Four days 5. Three days

6. Two days 7. One day 8. Less than one day 9. Never

1. How many days a week did you snack after dinner in the past month?

1. Everyday 2. Six days 3. Five days 4. Four days 5. Three days

6. Two days 7. One day 8. Less than one day 9. Never

1. How many days a week did you have dinner alone in the past month?

1. Everyday 2. Six days 3. Five days 4. Four days 5. Three days

6. Two days 7. One day 8. Less than one day 9. Never

1. Have you joined any sports club in or off campus except physical education class?

1. Yes 2. No

1. **a.** Do you have a habit of exercise for over thirty minutes per day and three days per week? (Excluding walking)

1. Yes（For years months） 2. No

**b**. If you choose［No］, please give your reasons (multiple choice)

1. Lack of time 2. Poor physical conditions 3. Non-confident

4. Lack of site 5. Lack of companion 6. Lack of instruction

7. Lack of money 8. Lack of interest 9. No reason

1. Does your family have a plan for sports spending?

1. Yes 2. No

1. Do your family members have a habit of exercises (except yourself)

1. Yes 2. No

1. How about your performance of major courses in last semester (or give your comprehensive performance if you have not attended major courses yet)?

1. Excellent 2. Good 3. Pass 4. Fail

1. Physical activity

**1a**、 During the last 7 days, on how many days did you do **vigorous** physical activities like heavy lifting, digging, or fast bicycling. ?

◆　 days per week

◆　No vigorous physical activities → Skip to question 2a

**1b、** How much time in total did you usually spend on one of those day doing vigorous physical activities?

hours minutes

**2a、** During the last 7 days, on how many days did you do **moderate** physical activities like carrying light loads, bicycling at a regular pace, or doubles tennis? Do not include walking.

◆　 days per week

◆　No moderate physical activities → Skip to question 3a

**2b、** How much time in total did you usually spend on one of those day doing moderate physical activities?

hours minutes

**3a、** During the last 7 days, on how many days did you **walk** for at least 10 minutes at a time? This includes walking at work and at home, walking to travel from place to place, and any other walking that you did solely for recreation, sport, exercise or leisure.

◆　 days per week

◆　No walking → Skip to question 4

**3b、** How much time in total did you usually spend walking on one of those days?

hours minutes

**4、** During the last 7 days, how much time did you usually spend **sitting** on a week day?

hours minutes

1. Questions below are about your sleep.

a. How many hours did you usually sleep at night in the past month？

(1) <5 hours (2) 5-6 hours (3) 6-7 hours

(4) 7-8 hours (5) 8-9 hours (6) >9 hours

b. For how many days did you feel difficult to initiate and maintain sleep in the past month?

(1) < 1 day (2) 1-3 days (3) 4-7 days

(4) 8-15 days (5) ≥ 16 days

1. Please read each statement and decide how much of the time the statement describes how you have been feeling during the past several days.

|  | | A little of the time | Some of the time | Good part of the time | Most of the time |
| --- | --- | --- | --- | --- | --- |
| 1 | I feel down-hearted and blue |  |  |  |  |
| 2 | Morning is when I feel the best |  |  |  |  |
| 3 | I have crying spells or feel like it |  |  |  |  |
| 4 | I have trouble sleeping at night |  |  |  |  |
| 5 | I eat as much as I used to |  |  |  |  |
| 6 | I still enjoy sex |  |  |  |  |
| 7 | I notice that I am losing weight |  |  |  |  |
| 8 | I have trouble with constipation |  |  |  |  |
| 9 | My heart beats faster than usual |  |  |  |  |
| 10 | I get tired for no reason |  |  |  |  |
| 11 | My mind is as clear as it used to be |  |  |  |  |
| 12 | I find it easy to do the things I used to |  |  |  |  |
| 13 | I am restless and cannot keep still |  |  |  |  |
| 14 | I feel hopeful about the future |  |  |  |  |
| 15 | I am more irritable than usual |  |  |  |  |
| 16 | I find it easy to make decisions |  |  |  |  |
| 17 | I feel that I am useful and needed |  |  |  |  |
| 18 | My life is pretty full |  |  |  |  |
| 19 | I feel that others would be better off if I were dead |  |  |  |  |
| 20 | I still enjoy the things I used to do |  |  |  |  |

1. Do you feel pressured with your studies?

1. No 2. Slight 3. Serious

1. Do you have a goal to strive for?

1. Yes 2. No

1. Do you think your life is meaningful?

1. Yes 2. No 3. I do not know

1. Have you ever thought of suicide?

1. Never 2. Once 3. More than once

1. Have you ever attempted suicide?

1. Never 2. Once 3. More than once

1. Do you think you live happily?

1. Yes 2. No

1. How many times have you had a cold in the past year？

1. No 2. Once 3. More than once

- Results of physical fitness measurement.

|  | Male | Female |
| --- | --- | --- |
| Hight（cm） |  |  |
| Weight（kg） |  |  |
| Vital capacity（ml） |  |  |
| 50 meter sprint（sec） |  |  |
| Standing broad jump（m） |  |  |
| Sit and reach（cm） |  |  |
| 1000 meter running（M） |  |  |
| 800 meter running（F） |  |  |
| Pull-up（M） |  |  |
| Sit-up（F） |  |  |
| Grip strength (kg) |  |  |
